# Soluble Expression and Characterization of Biologically Active* Bacillus anthracis* Protective Antigen in* Escherichia coli*

**DOI:** 10.1155/2016/4732791

**Published:** 2016-02-07

**Authors:** Nagendra Suryanarayana, Vanlalhmuaka, Bharti Mankere, Monika Verma, Kulanthaivel Thavachelvam, Urmil Tuteja

**Affiliations:** ^1^Microbiology Division, Defence Research and Development Establishment, Jhansi Road, Gwalior 474002, India; ^2^Entomology Division, Defence Research Laboratory, Tezpur, Assam 784001, India

## Abstract

Bacillus anthracis secretory protein protective antigen (PA) is primary candidate for subunit vaccine against anthrax. Attempts to obtain large quantity of PA from* Escherichia coli* expression system often result in the formation of insoluble inclusion bodies. Therefore, it is always better to produce recombinant proteins in a soluble form. In the present study, we have obtained biologically active recombinant PA in small scale* E. coli* shake culture system using three different expression constructs. The PA gene was cloned in expression vectors bearing trc, T5, and T7 promoters and transformed into their respective* E. coli* hosts. The growth conditions were optimized to obtain maximum expression of PA in soluble form. The expression construct PA-pET32c in DE3-pLysS* E. coli* host resulted in a maximum production of soluble PA (15 mg L^−1^) compared to other combinations. Purified PA was subjected to trypsin digestion and binding assay with lethal factor to confirm the protein's functionality. Biological activity was confirmed by cytotoxicity assay on J774.1 cells. Balb/c mice were immunized with PA and the immunogenicity was tested by ELISA and toxin neutralization assay. This study highlights the expression of soluble and biologically active recombinant PA in larger quantity using simpler* E. coli* production platform.

## 1. Introduction

*Bacillus anthracis*, the causative agent of the disease anthrax, has two major virulence factors, one in the form of exotoxins and the other in the form of unique capsule, which are encoded by the plasmids pXO1 and pXO2, respectively [1, 2]. The capsule is made up of poly-D-*γ*-glutamic acid, a unique virulent structure that helps the bacterium to escape phagocytosis by macrophages inside the host. The exotoxins consist of three proteins, namely, protective antigen (PA), lethal factor (LF), and edema factor (EF) which together constitute the active anthrax toxins (AnTx) [3]. Basically, AnTx belongs to A-B toxin superfamily where the B moiety binds to the cell surface (in this case, PA) and assists in translocating the catalytic A moiety (LF or EF) inside the cell [4]. Individually, none of the proteins are toxic, but if PA combines with LF or EF, it results in the formation of lethal toxin (LeTx) or edema toxin (EdTx), respectively [5, 6]. PA is a four-domain protein and can bind to either of the host cell receptors, capillary morphogenesis gene 2 (CMG2) or tumor endothelial marker 8 (TEM8), through its C-terminal domain [7, 8]. Once bound to the receptor, PA is cleaved at the sequence -RKKR- by host furin-like proteases to generate receptor bound 63 kDa fragment (PA63) and a free 20 kDa fragment (PA20). The cleaved PA63 in its active state is oligomerized to form a pore like structure into which LF and EF can competitively bind with high affinity [9]. Once bound, LF/EF is translocated into the cell through receptor mediated endocytosis [10, 11]. EF is calmodulin dependent adenylate cyclases which can increase the cAMP level inside the cell disturbing the water homeostasis resulting in characteristic edema observed during cutaneous anthrax. EF also impairs cytokine signalling pathways and neutrophil functions [12]. LF is a highly specific zinc metalloproteases that can cleave the members of MAP kinase kinases (MAPKK) family at their N-terminus resulting in altered cell signalling, increased production of cytokines tumor necrosis factor-*α*, interleukin-1*β*, and nitric oxide. LF is also known to activate inflammasome complex and caspases-1 mediated cellular apoptosis [13–16].

In US, the FDA approved Anthrax Vaccine Adsorbed (AVA) as the only vaccine commercially available for use against anthrax. It is prepared by adsorbing the cell free culture supernatant of acapsular strain of* B. anthracis* V770-NP1-R on aluminium hydroxide gel [17, 18]. In UK, Anthrax Vaccine Precipitate (AVP) is in use which is alum precipitated cell free culture supernatant of Sterne strain 34F2 [19, 20]. Both AVA and AVP contain PA as major immunogen with trace amounts of LF and EF. Although AVA and AVP are effective, their undefined composition, batch to batch variation, extensive dosing regimen, and adverse immunological side effects have made way for the search of second-generation anthrax vaccine containing recombinant PA that is under developmental stage [21, 22]. These recombinant vaccines differ from their predecessors in that their composition will be defined, amenable to large scale production, and will be free from any adverse side effects. In case of large scale immunization of human population, huge quantities of biologically active recombinant PA will be required. For this purpose, a scalable purification process has been developed to generate recombinant PA in multigram quantities from recombinant* E. coli* [23]. Apart from* E. coli*, numerous other attempts to develop expression systems are based on variety of organisms including attenuated strains of* B. anthracis*,* B. subtilis*,* B. brevis*, and* Salmonella typhimurium* [21, 24–26]. However, these approaches are not simpler and often require multiple harsh purification steps which may result in reduced yield and stability [27, 28]. It is therefore always better to express a protein in a soluble form, as it can result in functionally active and biologically stable protein. Moreover, soluble proteins are easy to purify and reduce the extra downstream processing that is involved in purifying the denatured proteins and refolding them to make functionally active form. In addition to this, most of the denatured proteins are susceptible to precipitation and protease degradation [29, 30] during downstream processing.

In the present study, we have expressed recombinant soluble PA in* E. coli* expression system using three different expression vectors,* namely,* pPROEXHTa, pQE30, and pET32c, bearing* trc*, T5, and T7 promoters, respectively. Further, each vector construct was transformed into different* E. coli* expression hosts and the growth conditions of the transformants were optimized so as to obtain the maximum yield in soluble form. Purification was carried out using simple chromatographic techniques and the yield of protein was compared. Further, the soluble PA from maximum producing clone PA-pET32c-DE3-pLysS was characterized by trypsin digestion and LF binding assay to confirm its functionality. The biological activity was confirmed through macrophage cell protection assay using the PA immunized sera. This study highlights the straightforward production of large quantity of recombinant PA in its biologically active soluble form using an optimized* E. coli* vector-host combination system.

## 2. Materials and Methods

### 2.1. Bacterial Strains and Reagents

The bacterial strains used in this study are listed in Table 1. The expression vector pPROEXHTa was purchased from Invitogen (USA), pQE30 from Qiagen (Germany), and pET32c from Novagen (Germany). Luria-Bertani broth/agar for maintaining the expression hosts was obtained from Himedia (India). Activated protective antigen was procured form List Biological Laboratories (USA). All other chemicals were procured from Sigma Aldrich (India) unless specified.

### 2.2. PA Gene Amplification and Recombinant Plasmids Construction

All the primers (Table 2) used in this study were custom synthesized from Sigma Aldrich, India. The* PagA* gene sequence was retrieved from NCBI (GenBank accession number AF306782). The genomic DNA was isolated from the* B. anthracis* clinical isolate by conventional method and was used as a template for PCR reaction. The PCR reaction was carried out in a 25 *μ*L reaction containing 50 ng of template DNA, 0.1 *μ*mol L^−1^ primers, 1x supertaq complete buffer (2 mmol L^−1^ MgSO_4_), 0.2 mmol L^−1^ dNTPs, and 2.5 units of super* Taq* DNA polymerase (Ambion, USA). The PCR condition was as follows: initial denaturation of 94°C for 5 minutes, 30 rounds of 1 minute denaturation at 94°C, 1 minute annealing at 50°C, and 2 minutes of extension at 72°C followed by 10 minutes of final extension at 72°C. The PCR product was electrophoresed on 0.8% agarose gel and was extracted using Qiagen gel extraction kit. The PCR product along with expression vectors pPROEXHTa, pQE30, and pET32c was restriction digested with* Bam*HI/*Xho*I,* Bam*HI/*Kpn*I, and* Bam*HI/*Sal*I (Fermentas, USA) combination, respectively, and was followed by ligation to obtain PA-pPROEXHTa, PA-pQE30, and PA-pET32c recombinant plasmids. The recombinant plasmid PA-pPROEXHTa was transformed into* E. coli* DH5*α*, BL21-DE3, and DE3-pLysS, PA-pQE30 in* E. coli* M15 and XL-1 Blue, and PA-pET32c plasmid in* E. coli* BL21-DE3 and DE3-pLysS, respectively. A total of seven different host-vector combinations were obtained. After transformation, the positive clones were confirmed by restriction digestion of the isolated plasmid with the following enzymes: PA-pPROEXHTa with* Bam*HI/*Xho*I, PA-pQE30 with* Bam*HI/*Kpn*I, and* PA-pET32c* with* Bam*HI/*Sal*I combination.

### 2.3. Expression and Purification

To determine the maximum expression of PA from individual clones, preliminary expression was carried out at different temperatures and time periods. All the* E. coli* cells carrying the recombinant plasmids were grown in 5 mL Luria-Bertani broth tubes at 37°C till the OD_600_ reached 0.6. Isopropyl *β*-D-1-thiogalactopyranoside (IPTG) was added at the final concentration of 1 mmol L^−1^ and the clones were further grown at 37°C and 22°C temperature for 3 hours, for 5 hours, and overnight. The cells were harvested at 8000 g for 5 minutes and were analyzed on 10% sodium dodecyl sulphate polyacrylamide gel electrophoresis (SDS-PAGE). The temperature and time period at which particular clone showed maximum expression of PA were noted and the same conditions were applied for bulk production of PA. For purification, all the seven clones were inoculated in 100 mL LB medium. Purification under native condition was performed by immobilized metal ion affinity chromatography (IMAC) using nickel trinitriloacetic acid (Ni-NTA) resin followed by anion exchange chromatography using diethylaminoethanol (DEAE). Briefly, the harvested cells were suspended in lysis buffer (300 mmol L^−1^ sodium chloride, 50 mmol L^−1^ sodium dihydrogen phosphate, and 1 mg mL^−1^ lysozyme, pH 8.0). Following sonication on ice bath (300 watt, 50% burst/50% cooling), the lysate was centrifuged at 10,000 g for 10 minutes. The supernatant was collected and passed through a preequilibrated Ni-NTA agarose column. The matrix was washed with wash buffer (300 mmol L^−1^ sodium chloride, 50 mmol L^−1^ sodium dihydrogen phosphate, and 20 mmol L^−1^ Imidazole, pH 8.0) and finally protein was eluted with 250 mmol L^−1^ imidazole. All the eluted fractions were pooled and dialyzed against 10 mmol L^−1^ Tris, pH 8, and further loaded onto DEAE sepharose column. Gradients of elutions from 10 mmol L^−1^ to 500 mmol L^−1^ sodium chloride were carried out and the fractions showing the maximum peak absorbance at 280 nm were pooled, dialyzed in PBS, concentrated to 1 mL volume, quantified by Nanodrop (Thermo Fisher 2000C), and stored at −20°C until further use. For purification under denatured condition, the harvested cells were washed twice with lysis buffer before dissolving in urea buffer (8 mol L^−1^ urea, 100 mmol L^−1^ sodium dihydrogen phosphate, and 10 mmol L^−1^ Tris, pH 8). The lysate was centrifuged at 10,000 g and the supernatant was loaded onto the urea buffer preequilibrated Ni-NTA agarose column. Elution was carried out at pH 4.5 and the pooled elutes were dialyzed against two changes of PBS. The protein was concentrated to 1 mL volume, quantified by Nanodrop, and stored at −20°C until further use.

### 2.4. SDS PAGE and Western Blotting

To see the recombinant PA expression and purification profile of different clones, SDS PAGE was performed. Briefly, all the seven clones after IPTG induction were harvested and lysed in 2x SDS sample lysis buffer. For purified proteins, 5 *μ*L of each sample was mixed with equal volume of 2x SDS sample lysis buffer before loading onto 10% SDS PAGE. After electrophoresis, the gel was stained with coomassie brilliant blue and image was captured through Gel doc XR+ imager system (Biorad, USA). For western blotting, the soluble cell lysate from all the clones was electrophoresed on 10% SDS PAGE and the gel was transferred to nitrocellulose membrane using TE70XP semidry blot system (Hoefer Inc, USA). Following transfer, the membrane was blocked with 5% skim milk for 1 hour at room temperature and then probed with anti-PA monoclonal antibody at 1 : 1000 dilutions for 1 hour. After washing thrice with PBS-Tween and PBS, the membrane was incubated with anti-mouse IgG-HRP conjugate at 1 : 5000 dilutions and was finally developed with 3,3′-diaminobenzidine tetrahydrochloride (DAB) substrate.

### 2.5. Trypsin Digestion of PA Molecule

The soluble PA purified from PA-pET32c-DE3pLysS clones was tested for its proper conformation and functionality by incubating 5 *μ*g of PA with 5 ng of trypsin (PA: trypsin at 1000 : 1 ratio) for 30 minutes at room temperature following which the samples were electrophoresed on 12% SDS-PAGE and stained with coomassie brilliant blue.

### 2.6. Binding to LF and Native-PAGE

For the confirmation of functional activity of the expressed PA, 2 *μ*g of trypsin digested PA was incubated with 2 *μ*g of LF at room temperature for 30 minutes. The samples were run on 4% native polyacrylamide gel at 4°C and visualized by staining the gel with coomassie brilliant blue.

### 2.7. Animal Immunization

Six female Balb/c mice were procured from Institutional Animal Care Center, DRDE, and were provided with food and water* ad libitum*. Each mouse was immunized subcutaneously with 10 *μ*g PA adsorbed on aluminium hydroxide adjuvant on days 0, 14, and 28. Mice receiving only PBS were taken as control. All mice were bled prior to first immunization on day 0 and on 35th day. Serum was separated and stored at −20°C until further use.

### 2.8. Enzyme Linked Immunosorbent Assay

To test the reactivity of PA immunized sera, indirect ELISA was performed by coating 1 *μ*g mL^−1^ PA in ELISA plate overnight at 4°C. After blocking with 5% skim milk for one hour, serial twofold dilution of PA immunized sera starting from 1 : 1000 was added and incubated for one hour at room temperature. Anti-mouse IgG HRP was added and further incubated for one more hour and finally developed using orthophenylenediamine dihydrochloride (OPD) as substrate. The reaction was stopped using 2 N H_2_SO_4_ and the absorbance was recorded at 490 nm. The test was performed in triplicate and the data was represented as mean ± SD. Cut-off value for the assays was calculated as the mean absorbance (+2 SD) from sera of control group assayed at 1 : 100 dilutions. The endpoint IgG titers were calculated as reciprocal of the highest serum dilution giving an absorbance more than the cut-off.

### 2.9. Cell Culture

Mouse macrophage cell line J774.1 was procured from National centre for cell sciences, Pune, India and was maintained in high glucose Dulbecco's Modified Eagle's Medium (DMEM) containing 10% fetal bovine serum (FBS) and 100 U mL^−1^ penicillin and streptomycin as antibiotics. One day before the experiment 4 × 10^4^ cells were seeded in a 96 well plate and incubated at 37°C in presence of 5% humidified CO_2_. The next day spent media was replaced by a fresh medium containing gradient concentration of soluble PA and native PA of* B. anthracis* from List Biologicals (0.005 *μ*g mL^−1^ to 5 *μ*g mL^−1^) and was incubated at 37°C along with 0.125 *μ*g mL^−1^ LF. After 4 hours, 20 *μ*L of 5 mg mL^−1^ MTT (3-(4,5 dimethylthiazol-2 yl)-2,5 diphenyl tetrazolium bromide) was added to each well and further incubated for 30 minutes. The formazan crystals developed were dissolved in 100 *μ*L acidified isopropanol (25 *μ*L of concentrated HCl and 500 *μ*L 10% SDS in 10 mL 90% isopropanol) and the absorbance was measured using UV-Vis microplate reader (BioTek, Winooski, VT) at 570 nm. Percentage viability was calculated by considering the well with no toxins as 100% survival. All the experiments were performed in triplicate and repeated at least three times. For toxin neutralization assay, J774.1 cells were seeded overnight as mentioned above. The next day, spent media were replaced with the medium containing 0.3 *μ*g mL^−1^ of PA and 0.125 *μ*g mL^−1^ of LF with twofold serial dilution of PA antiserum starting from 1 : 100. After incubating the plate for 5 hours, 20 *μ*L of 5 mg mL^−1^ MTT was added to each well and further incubated for 30 minutes. The formazan crystals developed were dissolved in 100 *μ*L acidified isopropanol and the absorbance was measured from UV-Vis microplate reader (BioTek, Winooski, VT) at 540 nm with reference wavelength at 640 nm. Wells containing only medium but no cells were taken as blank while the wells containing the cells with medium only were taken as positive control. Percentage viability was calculated as follows:

(1) $$\begin{array}{c} \left(\;\frac{A_{sample}-A_{blank}}{A_{pc}-A_{blank}}\;\right)\times 100, \end{array}$$

where *A*_sample_ is absorbance of test samples, *A*_blank_ is absorbance of blank, and *A*_pc_ is absorbance of positive control.

All the tests were performed in triplicate and repeated at least three times. The neutralization titer was defined as the lowest serum dilution that kills 50% of the macrophage cells.

## 3. Results

### 3.1. Construction of Recombinant Plasmids

**Figure 1:**
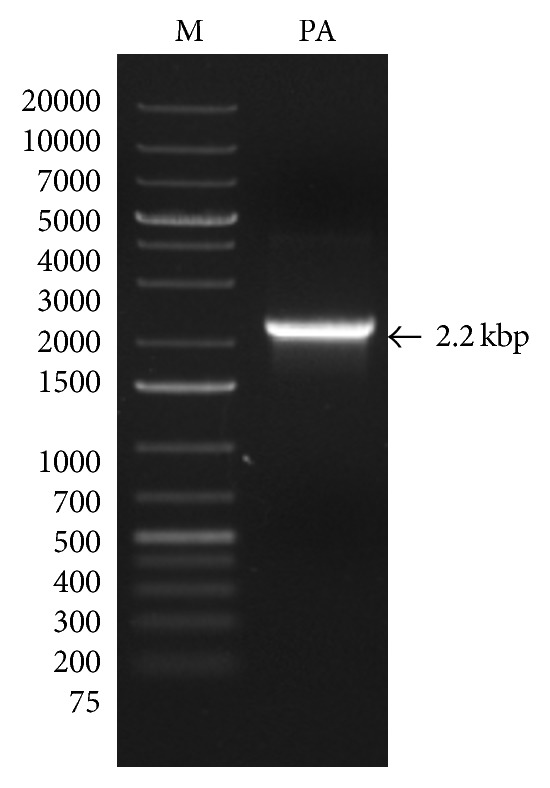
PCR amplification of mature PA gene. The PCR product was electrophoresed on 0.8% agarose gel and visualized under ultra violet lamp. M, DNA ladder; PA, protective antigen.

**Figure 2:**
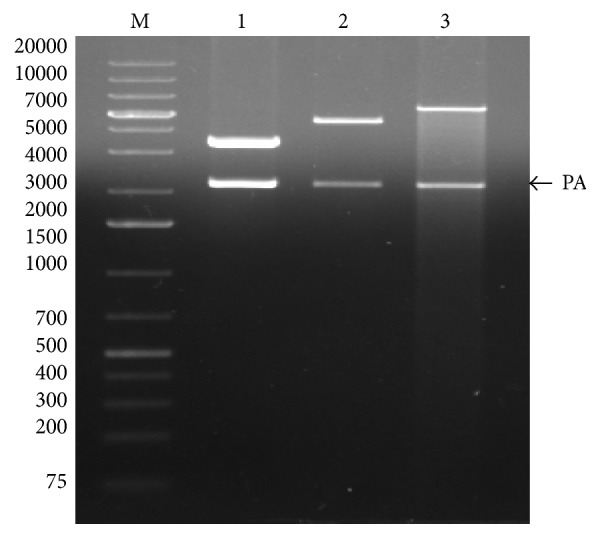
Restriction digestion of three different PA constructs. M, DNA marker; lane 1, PA-pQE30 digested with restriction enzymes* Bam*HI/*Kpn*I; lane 2, PA-pPROEXHTa digested with restriction enzymes* Bam*HI/*Xho*I; lane 3, PA-pET32c digested with restriction enzymes* Bam*HI/*Sal*I.

A 2.23-kilo base pair structural gene for PA was PCR amplified (Figure 1) and was successfully inserted into the plasmids pPROEXHTa, pQE30, and pET32c by restriction digestion and ligation. The recombinant plasmid PA-pPROEXHTa was transformed into* E. coli* hosts DH5*α*, BL21-DE3, and DE3-pLysS, PA-pQE30 into M15 and XL-1 Blue* E. coli* host, and PA-pET32c into BL21-DE3 and DE3-pLysS. The clones were confirmed by DNA sequencing and restriction digestion analysis of the isolated plasmid which released a 2.2 kb insert from all the three expression vectors (Figure 2).

### 3.2. Expression and Purification of PA

Since no differences were observed by changing the IPTG concentration, the expression studies were carried out with 1 mM IPTG. After induction, the clones were incubated for 3 hours, for 5 hours, and overnight at 37°C and 22°C. For each vector construct and host combination, optimal expression conditions were selected at which recombinant PA production was maximum. For PA-pQE30 in M15, PA-pPROEXHTa in DH5*α*, and BL21-DE3, the growth condition was maintained at 37°C for 5 hours after induction. The PA-pET32c construct in DE3-pLysS was grown at 22°C for 5 hours while PA-pQE30 in XL-1 Blue and PA-pET32c in BL21-DE3 were grown at 22°C overnight after IPTG induction. The purification was carried out under both native and denaturing condition. Among the seven clones, the highest yield of soluble PA, 15 mg L^−1^, was observed for pET32c construct in DE3-pLysS host. A pQE30 construct in XL-1 blue and M15 host produced 12 mg L^−1^ and 7.5 mg L^−1^ of recombinant PA. The pPROEXHTa harboring clone PA-pPROEXHTa in BL21-DE3 also yielded high amount of soluble recombinant PA at the range of 5.1 mg L^−1^. Comparatively, all the other clones produced less amount of soluble recombinant PA. The PA-pPROEXHTa construct in DH5*α* and DE3-pLysS produced 1.2 and 3 mg L^−1^ of soluble protein while the PA-pET32c construct in BL21-DE3 produced 1.5 mg L^−1^ of soluble PA. Apart from the soluble fractions, all the clones except PA-pQE30 in XL-1 blue yielded high amount of recombinant PA in denatured form. The purified proteins were dialyzed against PBS, concentrated to 1 mL volume, and stored in −20°C until further use. The total yields of soluble and denatured recombinant PA from all the seven clones are outlined in Table 3.

### 3.3. SDS PAGE and Western Blotting

**Figure 3:**
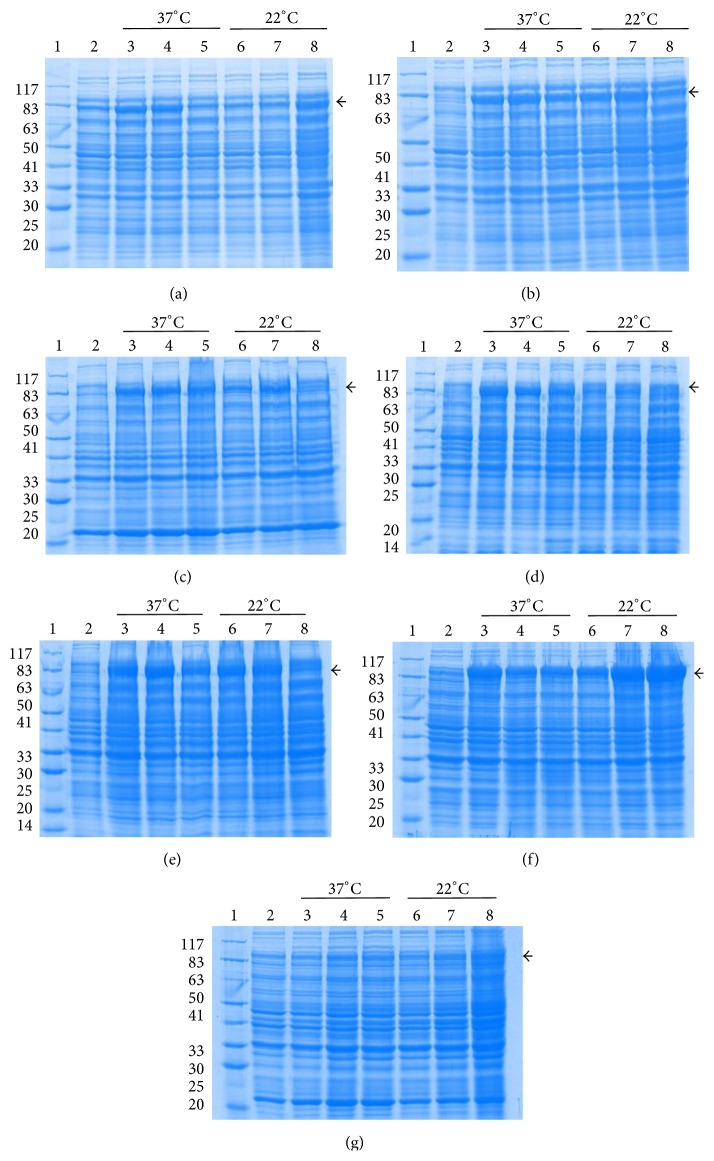
SDS-PAGE analysis of recombinant PA expression from seven different host-vector combinations at different temperature and time interval. Arrow marks indicate recombinant PA expression. (a) PA-pQE30-M15, (b) PA-pQE30-XL-1 blue, (c) PA-pPROEXHTa-DH5*α*, (d) PA-pPROEXHTa-BL21-DE3, (e) PA-pPROEXHTa-DE3-pLysS, (f) PA-pET32c-BL21-DE3, and (g) PA-pET32c-DE3-pLysS. Lane 1, protein molecular weight marker; lane 2, uninduced fractions; lanes 3, 4, and 5, post-IPTG growth for 3 hours, for 5 hours, and overnight, respectively, at 37°C temperature; lanes 6, 7, and 8, post-IPTG growth for 3 hours, for 5 hours, and overnight, respectively, at 22°C temperature.

**Figure 4:**
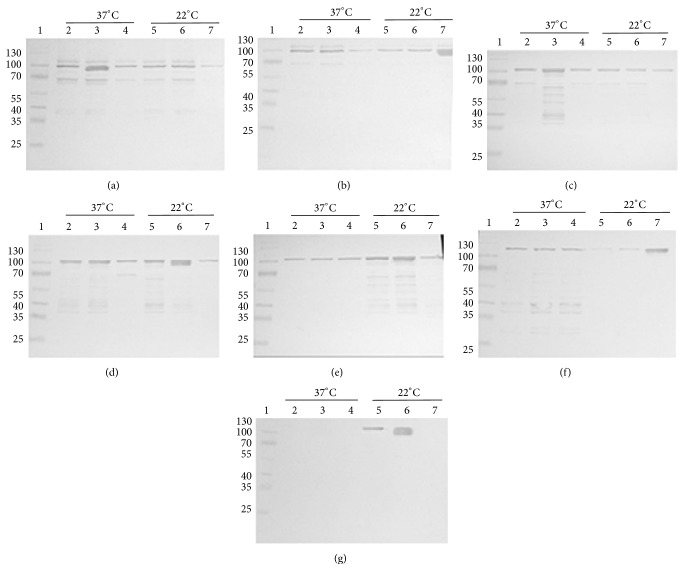
Western blotting analysis of soluble cell lysate from seven different PA expressing clones grown at different temperature and time intervals. (a) PA-pQE30-M15, (b) PA-pQE30-XL-1 blue, (c) PA-pPROEXHTa-DH5*α*, (d) PA-pPROEXHTa-BL21-DE3, (e) PA-pPROEXHTa-DE3-pLysS, (f) PA-pET32c-BL21-DE3, and (g) PA-pET32c-DE3-pLysS. Lane 1, protein molecular weight marker; lanes 2, 3, and 4, soluble cell lysate from 3 hours, 5 hours, and overnight culture, respectively, at 37°C temperature; lanes 5, 6, and 7, soluble cell lysate from 3 hours, 5 hours, and overnight culture, respectively, at 22°C temperature.

**Figure 5:**
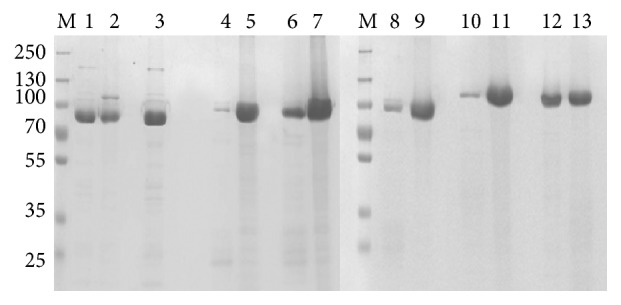
SDS-PAGE analysis of purified protein from soluble and denatured fractions of all the seven clones. 5 *μ*L of each fraction was mixed with 5 *μ*L 2x SDS sample lysis buffer and was loaded onto the gel and electrophoresed. M, protein molecular weight marker; lanes 1, 3, 4, 6, 8, 10, and 12, purified soluble PA protein from pQE30-M15, pQE30-XL-1 blue, pPROEXHTa-DH5*α*, pPROEXHTa-BL21-DE3, pPROEXHTa-DE3-pLysS, pET32c-BL21-DE3, and pET32c-DE3-pLysS, respectively; lanes 2, 5, 7, 9, 11, and 13, purified denatured PA protein from pQE30-M15, pQE30-XL-1 blue, pPROEXHTa-DH5*α*, pPROEXHTa-BL21-DE3, pPROEXHTa-DE3-pLysS, pET32c-BL21-DE3, and pET32c-DE3-pLysS, respectively.

**Figure 6:**
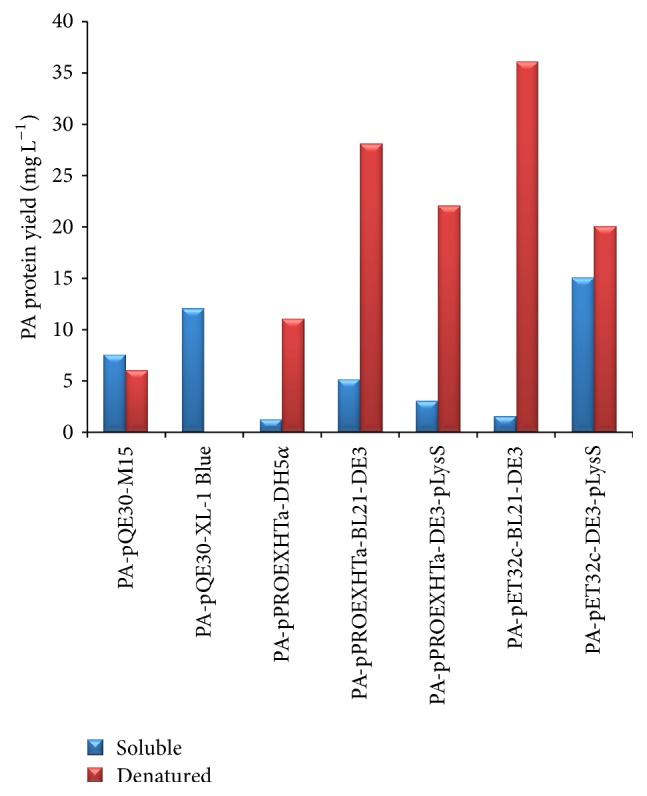
Graphical representation of total yield of recombinant PA expressed in soluble and denatured form. The total yield is represented in mg L^−1^. Blue bar represents total yield of soluble recombinant PA while red bar represents the total yield of denatured form of recombinant PA.

Expression profile of the induced samples and purified recombinant PA was analyzed on 10% SDS PAGE. All the seven clones expressed at different growth parameters and time intervals were loaded onto the gel (Figure 3). To determine the optimal growth conditions (temperatures and time periods) of each of the seven clones for maximum yield of soluble recombinant PA, western blotting with densitometric analysis was performed. Following SDS-PAGE, the soluble cell lysate of each clone was transferred onto nitrocellulose membrane and incubated with anti-PA monoclonal antibody. All the PA producing clones reacted to anti-PA monoclonal antibody (Figure 4). After purifying the proteins in soluble and denatured form, 5 *μ*L of the purified protein sample from each of the vector host combinations was electrophoresed on SDS-PAGE (Figure 5). The relative molecular weights of PA expressed along with their respective tags were 83 kDa in pQE30, 86 kDa in pPROEXHTa, and 100 kDa in pET32c. The total yield of recombinant PA in both soluble and denatured form has been depicted in graph (Figure 6).

### 3.4. Trypsin Digestion and Native-PAGE

**Figure 7:**
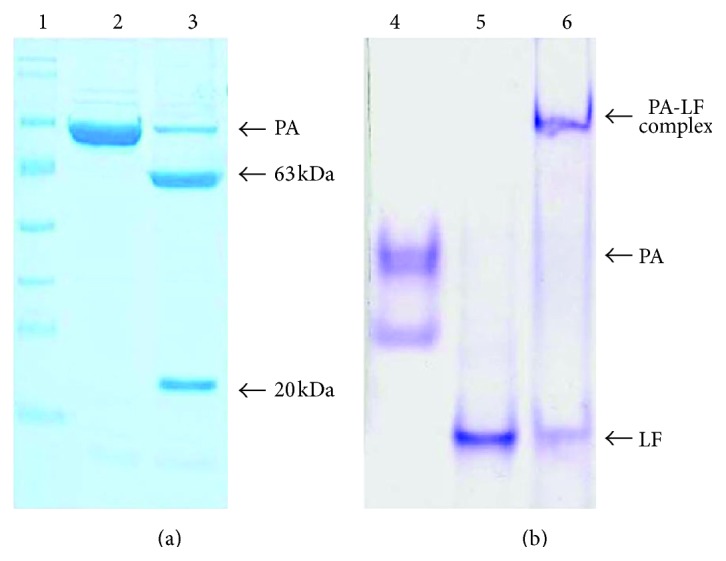
Trypsin digestion and native-PAGE analysis. (a) 5 *μ*g of PA was digested with 5 ng of trypsin for 30 minutes at room temperature and loaded onto 12% SDS-PAGE. 1, protein molecular weight marker; 2, 83 kDa recombinant PA; 3, trypsin digested PA showing two fragments 63 kDa and 20 kDa, respectively. Note that the 20 kDa fragment is around 33 kDa owing to the extra sequences at N-terminal end from the expression vector pET32c. (b) 2 *μ*g of trypsin digested PA was incubated with 2 *μ*g LF at room temperature for 30 minutes and electrophoresed on 4% native-PAGE. 4, PA; 5, LF; 6, PA-LF toxin complex.

Since PA from pET32c-DE3-pLysS was produced in soluble form in large quantity, the same was considered for further characterization. Trypsin digestion of PA resulted in the formation of two fragments of 20 kDa and 63 kDa (Figure 7(a)). For binding assay, trypsin digested PA was incubated with LF for 30 minutes at room temperature and electrophoresed on 4% native-PAGE. Binding of LF to PA was confirmed by the shift in mobility as compared to PA and LF alone (Figure 7(b)).

### 3.5. ELISA

**Figure 8:**
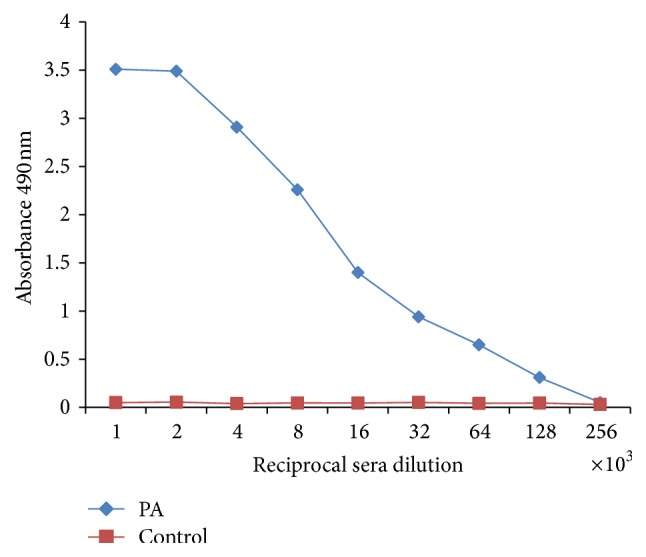
Indirect ELISA of PA immunized mice sera. ELISA plates were coated with 1 *μ*g mL^−1^ PA overnight at 4°C. Following blocking with 5% skimmed milk, the plates were incubated for one hour with serial twofold dilution of PA immunized mice sera starting from 1 : 1000 at room temperature. Plates were washed thoroughly and, after revealing with anti-mouse IgG HRP conjugate, the plates were developed by OPD. PA sera showed high antibody titer of 1.3 × 10^5^.

The IgG antibody titer was determined for PA immunized serum by Indirect ELISA. The 35th day serum was used for the assay. The reciprocal serum dilution was found to be 1.35 × 10^5^ for 35th day, explaining the high immunogenicity of immunized sera against recombinant PA (Figure 8).

### 3.6. Biological Activity

**Figure 9:**
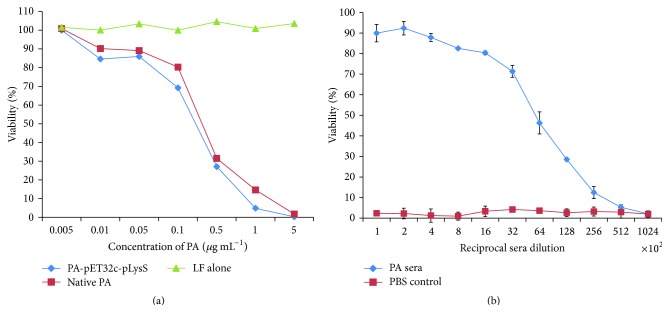
Biological activity of PA antigen and its mice immunized sera on J774.1 macrophage cells. (a) Cytotoxicity of recombinant PA in comparison to native PA from* Bacillus anthracis* when incubated along with 0.125 *μ*g mL^−1^ LF. Cells containing LF alone were taken as negative control. Recombinant PA showed 50% cytotoxicity at 0.3 *μ*g mL^−1^ while native PA showed 0.25 *μ*g mL^−1^. (b) Toxin neutralization of PA immunized sera. J774.1 cells were treated with 0.3 *μ*g mL^−1^ of PA, 0.25 *μ*g mL^−1^ of LF, and serial twofold dilution of PA immunized mice sera starting from 1 : 100. After 5 hours of incubation, 20 *μ*L of 5 mg mL^−1^ MTT dye was added, and after 30 minutes, the formazan crystals developed were dissolved by acidified isopropanol. The toxin neutralization titer was defined as the highest sera dilution showing 50% death of macrophage. For PA sera, it was found to be 6.4 × 10^3^.

To determine the biological activity of soluble PA, MTT based cytotoxicity and toxin neutralization assay was performed. Recombinant soluble PA protein was found to be lethal for the growth of macrophage cell lines with 50% viability around 0.3 *μ*g mL^−1^ (Figure 9(a)). Native PA also showed the similar cytotoxicity at the concentration 0.25 *μ*g mL^−1^, thereby confirming that the recombinant PA works equivalent to that of native PA. PA immunized sera showed robust toxin neutralization ability as determined by MTT assay. The toxin neutralization titer was found to be 6.4 × 10^3^ (Figure 9(b)).

## 4. Discussion

PA, the major component of* B. anthracis* tripartite toxin, is produced and secreted by the vegetative form of bacterium inside the host and recently it was reported to be present in trace amounts on its spore surfaces [31]. Eventually, the PA protein is the main component of commercial anthrax vaccine formulation and also the targeted molecule of various anthrax diagnostic assays. Due to the risk associated with the handling of* B. anthracis* culture for production and purification of native PA from bacterial culture supernatant, various laboratories have opted for the production of recombinant PA expressed conveniently in* E. coli* host. Although production of recombinant antigen as a heterologous protein in* E. coli* system is a well opted strategy, the suitable vector-host combination and the optimal growth condition to obtain maximal yield of functionally active protein are always empirical. In the present study, we have optimized the growth parameters for* E. coli* to obtain a maximum production of recombinant PA in soluble form from three different expression vectors. Initially, the gene sequence coding for matured PA protein was amplified from* B. anthracis* and cloned into three different expression vectors pPROEXHTa, pQE30, and pET32c. These constructs were transformed to their respective hosts and were grown at optimized temperature and time period after IPTG induction. Owing to the hurdles experienced during the downstream processing of the inclusion bodies like solubilization and proper refolding of denatured proteins, we focused our interest solely on the soluble expression of PA. The PA-pET32c construct produced higher recombinant PA in soluble form (15 mg mL^−1^) when expressed in* E. coli* DE3-pLysS. PA-pQE30 construct in M15 and XL-1 blue produced 7.5 and 12 mg mL^−1^ of soluble recombinant PA. Clones in pPROEXHTa were initially expected to yield lower level of the recombinant protein owing to the* trc* promoters as compared to the stronger T7 and T5 promoters. However, when transformed with BL21-DE3* E. coli* strains as host, the expression of soluble PA was 5.1 mg mL^−1^. In comparison to the high expressing clones mentioned above, the PA-pET32c construct in BL21-DE3 and PA-pPROEXHTa construct in DH5*α* and BL21-DE3 resulted in low level of soluble expression at 1.5 mg mL^−1^, 1.2 mg mL^−1^, and 3 mg mL^−1^, respectively. The low levels of soluble PA expression in these clones were accompanied by high expression of PA as inclusion bodies. Although T7 promoter has been known as a very strong promoter [32] occasionally, very high level of expression inside a bacterial host leads to the formation of cytoplasmic inclusion bodies which are insoluble and nonfunctional [33, 34]. Proper folding and biological activity of the recombinant proteins was confirmed by digestion with trypsin which produced a 63 kDa active protein followed by binding assay with LF. PA from pET32c-BL21-DE3-pLysS clone resulted in two fragments after trypsin digestion and could bind to LF as determined by native-PAGE. These functional activities were not observed with any of the resolubilized and refolded inclusion bodies from any of the constructs, as trypsin digestion resulted in the complete degradation which highlights the need for a functionally active PA protein (data not provided). Further trypsin digested PA could bind to LF in solution and resulted in anthrax lethal toxin complex as determined by the shift in mobility in native-PAGE. These experiments proved that the expressed PA was functionally active. To determine the biological activity of purified recombinant PA, cytotoxicity and toxin neutralization from PA immunized sera were performed on mouse macrophage cell J774.1. Cytotoxicity of the PA protein on macrophage cells was found to be 0.3 *μ*g mL^−1^ which was almost equivalent to that of the native protein. Toxin neutralization of PA immunized sera was robust with the neutralization titer of 6.4 × 10^3^. Thus, the present study describes the production of biologically active soluble recombinant PA from* E. coli* expression system by simple purification steps without need for complicated downstream processing as reported by earlier studies [35–37]. Multigram quantities of soluble recombinant PA can be obtained by further employing scale-up facilities for the above procedure. Therefore, these expression systems can be exploited for the production of cost-effective recombinant vaccine molecule against the deadly disease anthrax.

## Figures and Tables

**Table 1:** Bacterial strains used in this study.

Bacterial strains	Source
*Bacillus anthracis* clinical isolate	I.V.P.M^*∗*^, Vellore, India
DH5*α*	Invitrogen, USA
BL21-DE3, DE3-pLysS	EMD4 Biosciences, Germany
M15	Qiagen, Germany
XL-1 blue	Stratagene, USA

^*∗*^Institute of Veterinary and Preventive Medicine.

**Table 2:** Primers used in this study.

Primers	5′ to 3′ sequence	Restriction enzymes
pPROEXHTa_F	AGAGAAGGATCCAGAAGTTAAACAGGAGA	*Bam*HI
pPROEXHTa_R	CATCTCACTCGAGTTATCCTATCTCATAGCCT	*Xho*I
pQE30_F	GCGCAGGCCGGATCCGAAGTTAAA CAG	*BamHI*
pQE30_R	CCTAGAGGTACCTTATCC	*KpnI*
pET 32c_F	GAGAAGGATCCGAATGGAAGTTAAACAGGAGA	*Bam*HI
pET32c_R	CATCTCAGTCGACTTATCCTATCTCATAGCCT	*Sal*I

Sequence underlined represents restriction sites.

**Table 3:** Optimum growth conditions and total yield of recombinant PA protein from different host-vector combinations.

Sl. number	Clones	Optimal growth condition at 1 mmol L^−1^ IPTG	Wet pellet weight of one-litre culture	Total yield of soluble protein per litre of culture volume	Total yield of denatured protein per litre of culture volume
1	PA-pQE30-M15	37°C, 5 hours	6.17 g	7.5 mg	6.0 mg
2	PA-pQE30-XL-1 Blue	22°C, overnight	5.49 g	12.0 mg	—
3	PA-pPROEXHTa-DH5*α*	37°C, 5 hours	6.69 g	1.2 mg	11.0 mg
4	PA-pPROEXHTa-BL21 (DE3)	37°C, 5 hours	4.15 g	5.1 mg	28.0 mg
5	PA-pPROEXHTa-DE3-pLysS	22°C, 5 hours	2.94 g	3.0 mg	22.0 mg
6	PA-pET32c-BL21-DE3	22°C, overnight	5.72 g	1.5 mg	36.0 mg
7	PA-pET32c-DE3-pLysS	22°C, 5 hours	6.32 g	15.0 mg	20.0 mg

## References

[1] Mikesell P., Ivins B. E., Ristroph J. D., Dreier T. M. (1983). Evidence for plasmid-mediated toxin production in *Bacillus anthracis*. *Infection and Immunity*.

[2] Green B. D., Battisti L., Koehler T. M., Thorne C. B., Ivins B. E. (1985). Demonstration of a capsule plasmid in *Bacillus anthracis*. *Infection and Immunity*.

[3] Young J. A. T., Collier R. J. (2007). Anthrax toxin: receptor binding, internalization, pore formation, and translocation. *Annual Review of Biochemistry*.

[4] Mogridge J., Cunningham K., Lacy D. B., Mourez M., Collier R. J. (2002). The lethal and edema factors of anthrax toxin bind only to oligomeric forms of the protective antigen. *Proceedings of the National Academy of Sciences of the United States of America*.

[5] Leppla S. H. (1982). Anthrax toxin edema factor: a bacterial adenylate cyclase that increases cyclic AMP concentrations of eukaryotic cells. *Proceedings of the National Academy of Sciences of the United States of America*.

[6] Deusbery N. S., Webb C. P., Leppla S. H. (1998). Proteolytic inactivation of MAP-kinase-kinase by anthrax lethal factor. *Science*.

[7] Petosa C., Collier R. J., Klimpel K. R., Leppla S. H., Liddington R. C. (1997). Crystal structure of the anthrax toxin protective antigen. *Nature*.

[8] Bradley K. A., Mogridge J., Mourez M., Collier R. J., Young J. A. T. (2001). Identification of the cellular receptor for anthrax toxin. *Nature*.

[9] Scobie H. M., Rainey G. J. A., Bradley K. A., Young J. A. T. (2003). Human capillary morphogenesis protein 2 functions as an anthrax toxin receptor. *Proceedings of the National Academy of Sciences of the United States of America*.

[10] Klimpel K. R., Molloy S. S., Thomas G., Leppla S. H. (1992). Anthrax toxin protective antigen is activated by a cell surface protease with the sequence specificity and catalytic properties of furin. *Proceedings of the National Academy of Sciences of the United States of America*.

[11] Abrami L., Liu S., Cosson P., Leppla S. H., van der Goot F. G. (2003). Anthrax toxin triggers endocytosis of its receptor via lipid raft mediated clathrin dependent process. *Journal of Chemical Biology*.

[12] Szarowicz S. E., During R. L., Li W., Quinn C. P., Tang W.-J., Southwick F. S. (2009). *Bacillus anthracis* edema toxin impairs neutrophil actin based motility. *Infection and Immunity*.

[13] Friedlander A. M. (1986). Macrophages are sensitive to anthrax lethal toxins through an acid dependent process. *The Journal of Biological Chemistry*.

[14] Friedlander A. M., Bhatnagar R., Leppla S. H., Johnson L., Singh Y. (1993). Characterization of macrophage sensitivity and resistance to anthrax lethal toxin. *Infection and Immunity*.

[15] Hanna P. C., Kochi S., Collier R. J. (1992). Biochemical and physiological changes induced by anthrax lethal toxin in J774 macrophage-like cells. *Molecular Biology of the Cell*.

[16] Newman Z. L., Crown D., Leppla S. H., Moayeri M. (2010). Anthrax lethal toxin activates the inflammasome in sensitive rat macrophages. *Biochemical and Biophysical Research Communications*.

[17] Ivins B. E., Ezzell J. W., Jemski J., Hedlund K. W., Ristroph J. D., Leppla S. H. (1986). Immunization studies with attenuated strains of *Bacillus anthracis*. *Infection and Immunity*.

[18] Ivins B., Fellows P. F., Nelson G. O. (1994). Efficacy of standard human anthrax vaccine against *Bacillus anthracis* spore challenge in guinea pigs. *Vaccine*.

[19] Turnbull P. C. B. (1991). Anthrax vaccines: past, present and future. *Vaccine*.

[20] Brey R. N. (2005). Molecular basis for improved anthrax vaccines. *Advanced Drug Delivery Reviews*.

[21] Baillie L. W. J. (2006). Past, imminent and future human medical countermeasures for anthrax. *Journal of Applied Microbiology*.

[22] Bouzianas D. G. (2007). Potential biological targets of *Bacillus anthracis* in anti-infective approaches against the threat of bioterrorism. *Expert Review of Anti-Infective Therapy*.

[23] Chauhan V., Singh A., Waheed S. M., Singh S., Bhatnagar R. (2001). Constitutive expression of protective antigen gene of *Bacillus anthracis* in *Escherichia coil*. *Biochemical and Biophysical Research Communications*.

[24] Coulson N. M., Fulop M., Titball R. W. (1994). Bacillus anthracis protective antigen, expressed in *Salmonella typhimurium* SL 3261, affords protection against anthrax spore challenge. *Vaccine*.

[25] Ramirez D. M., Leppla S. H., Schneerson R., Shiloach J. (2002). Production, recovery and immunogenicity of the protective antigen from a recombinant strain of *Bacillus anthracis*. *Journal of Industrial Microbiology and Biotechnology*.

[26] Rhie G.-E., Park Y.-M., Chun J.-H., Yoo C.-K., Seong W.-K., Oh H.-B. (2005). Expression and secretion of the protective antigen of *Bacillus anthracis* in *Bacillus brevis*. *FEMS Immunology and Medical Microbiology*.

[27] Rhie G.-E., Park Y.-M., Han J.-S., Yu J.-Y., Seong W.-K., Oh H.-B. (2005). Efficacy of non-toxic deletion mutants of protective antigen from *Bacillus anthracis*. *FEMS Immunology and Medical Microbiology*.

[28] Chang H.-H., Tsai M.-F., Chung C.-P. (2006). Single step purification of recombinant anthrax lethal factor from periplasm of *Escherichia coli*. *Journal of Biotechnology*.

[29] Singh S. M., Panda A. K. (2005). Solubilization and refolding of bacterial inclusion body proteins. *Journal of Bioscience and Bioengineering*.

[30] Yamaguchi H., Miyazaki M. (2014). Refolding techniques for recovering biologically active recombinant proteins from inclusion bodies. *Biomolecules*.

[31] Welkos S., Little S., Friedlander A., Fritz D., Fellows P. (2001). The role of antibodies to *Bacillus anthracis* and anthrax toxin components in inhibiting the early stages of infection by anthrax spores. *Microbiology*.

[32] Studier F. W., Moffatt B. A. (1986). Use of bacteriophage T7 RNA polymerase to direct selective high-level expression of cloned genes. *Journal of Molecular Biology*.

[33] Betts S., King J. (1999). There's a right way and a wrong way: in vivo and in vitro folding, misfolding and subunit assembly of the P22 tailspike. *Cell*.

[34] Terpe K. (2006). Overview of bacterial expression systems for heterologous protein production: from molecular and biochemical fundamentals to commercial systems. *Applied Microbiology and Biotechnology*.

[35] Gupta P., Waheed S. M., Bhatnagar R. (1999). Expression and purification of the recombinant protective antigen of *Bacillus anthracis*. *Protein Expression and Purification*.

[36] Sharma M., Swain P. K., Chopra A. P., Chaudhary V. K., Singh Y. (1996). Expression and purification of anthrax toxin protective antigen from *Escherichia coli*. *Protein Expression and Purification*.

[37] Miller J., McBride B. W., Manchee R. J., Moore P., Baillie L. W. J. (1998). Production and purification of recombinant protective antigen and protective efficacy against *Bacillus anthracis*. *Letters in Applied Microbiology*.

